# 高效金属有机骨架气相色谱固定相的理性设计

**DOI:** 10.3724/SP.J.1123.2023.05002

**Published:** 2023-10-08

**Authors:** Han YANG, Wenqi TANG, Chu ZENG, Shasha MENG, Ming XU

**Affiliations:** 南京师范大学化学与材料科学学院, 江苏 南京 210023; School of Chemistry and Materials Science, Nanjing Normal University, Nanjing 210023, China

**Keywords:** 金属有机骨架材料, 气相色谱, 固定相, 热力学相互作用, 动力学扩散, 综述, metal organic frameworks (MOFs), gas chromatography (GC), stationary phase, thermodynamic interaction, kinetic diffusion, review

## Abstract

金属有机骨架材料(MOFs)由金属离子或金属簇与有机配体组装而成,其中多变的金属中心和有机配体使其具有高度可调性,这为调控高效气相色谱分离性能奠定了良好的结构基础。热力学作用力是描述分析物与固定相相互作用的基本指标,保留因子、麦氏常数、焓变与熵变等热力学值可以反映热力学作用力的相对大小。在微观层面上,可以通过设计MOFs孔隙内的多元作用力以开展热力学性质的研究,如设计金属亲和性、*π-π*相互作用、极性、手性位点等,这些热力学作用力可为分离具有微小差异的分析物提供有利环境。在动力学方面,MOFs的孔径大小与形状、颗粒尺寸、堆积模式对分析物的动力学扩散速率有着重要的影响,从改善分析物的动力学扩散角度出发,通过选择合适的孔径尺寸与形状、降低MOFs的颗粒尺寸、调控MOFs的堆积模式等手段,均可以提高气相色谱固定相的分离性能。根据色谱动力学统一方程和范蒂姆特方程计算扩散系数、理论塔板高度等动力学值,可有效评价色谱峰峰形和色谱柱柱效。在分离过程中,分析物的热力学作用力和动力学效应是协同作用的,且缺一不可。因此,本文从热力学与动力学两个角度提出了构建高效MOFs气相色谱固定相的设计思路,希望能为相关领域的研究提供一定帮助。

## Abstract (en)

Metal organic frameworks (MOFs) are assembled from metal ions or clusters and organic ligands. The high tunability of these components offers a solid structural foundation for achieving efficient gas chromatography (GC) separation. This review demonstrates that the design of high performance MOFs with suitable stationarity should consider both the thermodynamic interactions provided by these MOFs and the kinetic diffusion of analytes. Thermodynamic parameters are basic indicators for describing the interactions between various analytes and the stationary phase. Thermodynamic parameters such as retention factors, McReynolds constants, enthalpy changes, and entropy changes can reflect the relative intensity of thermodynamic interactions. For example, a larger enthalpy change indicates a stronger thermodynamic interaction between the analytes and stationary phase, whereas a smaller enthalpy change indicates a weaker interaction. In addition, the degree of entropy change reflects the relative degrees of freedom of analytes in the stationary phase. A larger entropy change indicates that the analytes have fewer degrees of freedom in the stationary phase. The higher the degree of restriction, the closer the adsorption of the analytes and, thus, the longer the retention time. Thermodynamic interactions, such as metal affinity, *π-π* interactions, polarity, and chiral sites, can be rationally introduced into MOF structures by pre- or post-modifications depending on the target analytes. These tailored thermodynamic interactions create a favorable environment with subtle differences for efficient analyte separation. For example, MOF stationarity may require large conjugation centers to provide specific *π-π* interactions to separate benzenes. Chiral groups may be required in the MOF structure to provide sufficient interactions to separate chiral isomers. The kinetic diffusion rate of the analytes is another critical factor that affects the separation performance of MOFs. The diffusion coefficients of analytes in the stationary phase (*D*_s_) can be used to evaluate their diffusion rates. The chromatographic dynamics equation illustrates that the chromatographic peak of analytes tends to be sharper and more symmetrical when the *D*_s_ is large, whereas a wider trailing peak may appear when the *D*_s_ is small. The Van Deemter equation also proves that a low *D*_s_ may lead to a high theoretical plate height and low column efficiency, whereas a high *D*_s_ may lead to a low theoretical plate height and increased column efficiency. Analyte diffusion can be significantly influenced by the pore size, shape, particle size, and packing mode of MOFs. For instance, an excessively small pore size results in increased mass transfer resistance, which affects the diffusion of analytes in the stationary phase, probably leading to serious peak trailing. Thus, a suitable pore size is required to enhance the kinetic diffusion of analytes and improve the separation performance of MOFs. Theoretically, the design of a high performance MOF stationary phase requires the creation of routes for the rapid diffusion of analytes. However, the separation ability of an MOF is determined by not only the kinetic diffusion rate of the analytes but also the thermodynamic interactions it provides. An excessively fast diffusion rate may lead to insufficient interactions between the analytes and MOFs, compromising their ability to effectively separate different analytes. The thermodynamic interactions and kinetic diffusion of analytes are synergistic and mutually essential. Therefore, this review concludes with research on the influence of both the thermodynamic interactions and kinetic diffusion of analytes on the performance of MOF stationary phases. Based on the findings of this review, we propose that high performance MOF stationary phases can be achieved by balancing the thermodynamic interactions and kinetic diffusion of analytes in these phases through the rational design of the MOF structure. We believe that this review provides useful guidelines for the design of high performance MOF stationary phases.

气体分离具有十分重要的意义,例如烷烃异构体和苯系物的分离都被认为是改变世界的七大分离之一^[1]^。传统的化工气体分离常常以蒸馏、萃取等热分离方式进行,其经济成本高、分离时间长,且分离选择性低,如难以分离尺寸与沸点相近的二甲苯异构体^[2]^。相比之下,吸附分离具有更高的灵敏度与分辨率,可以实现不同气体成分的快速、高效分离,其中,气相色谱是较为常用的一种微量气体的分离技术,其设备易于操作,且对环境较为友好^[3]^。

气相色谱分离能力的核心是色谱固定相。目前已被报道的固定相有沸石类多孔材料、柔性聚合物、共价有机骨架、多孔氧化物、硫族化物、多孔配位聚合物和离子液体等^[4⇓⇓-7]^。金属有机骨架材料(MOFs)是由有机配体(多为芳香族多元羧酸)和金属离子或金属簇自组装而成的多孔晶态材料^[8⇓⇓⇓-12]^。MOFs具有较大的比表面积和孔隙率,这为气体的吸附和分离提供了丰富的活性位点和灵活的扩散路径。此外,多变的有机配体和金属中心使MOFs的形状与孔径具有高度可调性,可在原子水平上对其内部孔隙结构进行精确控制,从而可针对不同种类的目标物来设计合适的孔道结构。因此,MOFs被认为是非热分离^[13,14]^的首选材料之一,在气体分离过程中具有重要的意义^[15⇓⇓⇓-19]^。目前,MOFs作为气相色谱固定相已在各种混合气体的分离中得到了广泛应用,如烷烃、二甲苯异构体、有机污染物等^[20]^。

高效MOFs固定相的理性设计可以从调控分析物与MOFs之间的热力学相互作用力和分析物在MOFs孔道中的动力学扩散两方面出发(图1)。热力学参数是描述分析物与固定相之间相互作用力强弱的基本指标,而动力学扩散是推动实现色谱高效分离的有效因素。在微观尺度下,可通过设计MOFs孔道并引入不同的官能团来提供独特的作用力,以区分分析物之间的微小差别,最终实现分析物的高效分离。根据不同的目标物性质,可针对性地向MOFs引入金属亲和性、*π-π*相互作用、极性和手性位点等单元或多元作用力。从动力学扩散的角度出发,MOFs的孔道尺寸和形状为分析物的扩散提供了通路,因此根据所分离的目标物来调控孔道尺寸与形状能够有效调控动力学扩散路径与速率。例如,调控MOFs的颗粒尺寸和堆积模式等方式能够为分析物提供合适的扩散路径。此外,设计一个具有高效分离能力的色谱固定相应协同考虑MOFs所能提供的热力学作用力和适合分析物扩散的孔道环境。单一考虑热力学作用力所构建的MOFs固定相可能具备分离能力,但得不到窄而尖的色谱峰;单一考虑分析物的动力学扩散所构建的MOFs固定相可能获得窄而尖的色谱峰,但无法分开分析物,甚至对分析物不具备保留能力。本文总结了以下3点内容:(1)通过在MOFs中设计不同的热力学作用位点以改善色谱分离效果;(2)通过调控MOFs的颗粒尺寸、孔径、形状及堆积模式来加速分析物的动力学扩散,以改善固定相分离性能;(3)协同调控热力学作用力与动力学效应以实现高效分离,并获得独特的出峰顺序。

**图 1:**
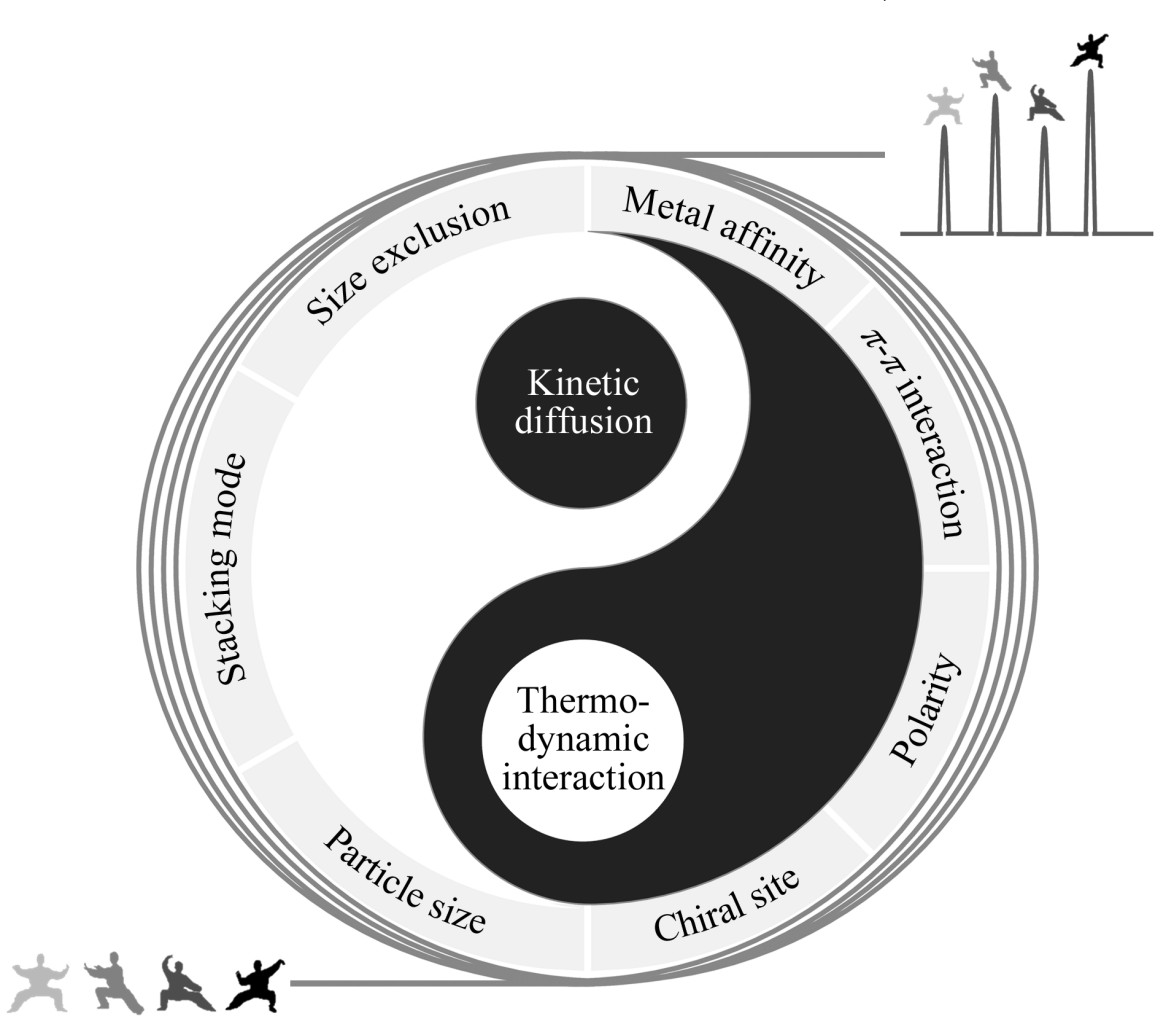
基于热力学与动力学效应的MOFs固定相气相色谱分离示意图

## 1 热力学相互作用

热力学相互作用是影响气相色谱分离效率的重要因素之一,其主要由固定相本身的性质以及其与分析物之间的相互作用决定。色谱基本方程、麦氏常数与范特霍夫方程分别从不同角度为热力学作用力的强弱提供了相应表征手段。色谱基本方程:

*t*_R_=*t*_0_(*k*+1)

*t*_R_和*t*_0_分别为保留时间(s)和死时间(s),保留因子(*k*)指分析物在流动相与固定相中达到分配平衡时的质量比,*k*值越大表示固定相对该分析物的容量越大,保留时间越长。结合范特霍夫方程可得到分析物的焓变和熵变:

ln *k*=- $\frac{\Delta H}{RT}$ + $\frac{\Delta S}{R}$ +ln *φ*

其中,Δ*H*为焓变(kJ/mol),Δ*S*为熵变(J/(mol·K),*R*为气体常数(J/(mol·K)), *T*为绝对温度(K), *φ*为相比(固定相与流动相的体积比)。通常Δ*H*的绝对值越大,表明分析物与固定相之间的相互作用越强,进而表现出更长的保留时间。此外,麦氏常数被广泛用于描述色谱柱的极性^[21]^。以苯、正丁醇、2-戊酮、硝基丙烷和吡啶为标准物质,在120 ℃的柱温下,分别测定它们在色谱柱和角鲨烷中的保留指数差值(Δ*I*),并将其作为特征常数,5个Δ*I*的平均值反映了色谱柱的极性大小,该数值越大,表明色谱柱的极性越大。针对MOFs高度可调的特点,通过增强热力学相互作用对其进行结构优化,如提供*π-π*相互作用、开放金属位点、手性位点等,可进一步加强分析物与MOFs固定相之间的相互作用,以拓展MOFs在气相色谱分离中的应用。

### 1.1 金属亲和性

MOFs中金属离子或金属簇的亲和性通常会影响其与分析物相互作用的强度^[22,23]^,进而影响分离效果。因此,通过选择和设计具有适当金属亲和性的MOFs固定相可以实现分析物的高效分离^[24]^。

2010年,Gu等^[25]^报道了MOFs涂覆毛细管色谱柱(MIL-101)用于4种二甲苯异构体的分离。孔径为2.9~3.4 nm的MIL-101^[26]^(图2a)具有开放的Cr金属位点、出色的化学稳定性和热稳定性,并展现出100 s内基线分离4种异构体的优异性能。为验证开放金属位点对色谱分离的影响,作者通过合成后修饰将吡啶配位至MIL-101的开放金属位点上,经过相同的动态涂覆后,获得了吡啶修饰的MIL-101色谱柱。在分离二甲苯异构体时,吡啶修饰的MIL-101色谱柱没有展现出对二甲苯与间二甲苯的基线分离能力(图2b、2c),这是因为吡啶与开放金属位点间的配位减少了MIL-101固定相金属位点与异构体之间的电子供体和受体相互作用,进而降低了分离选择性。

**图 2:**
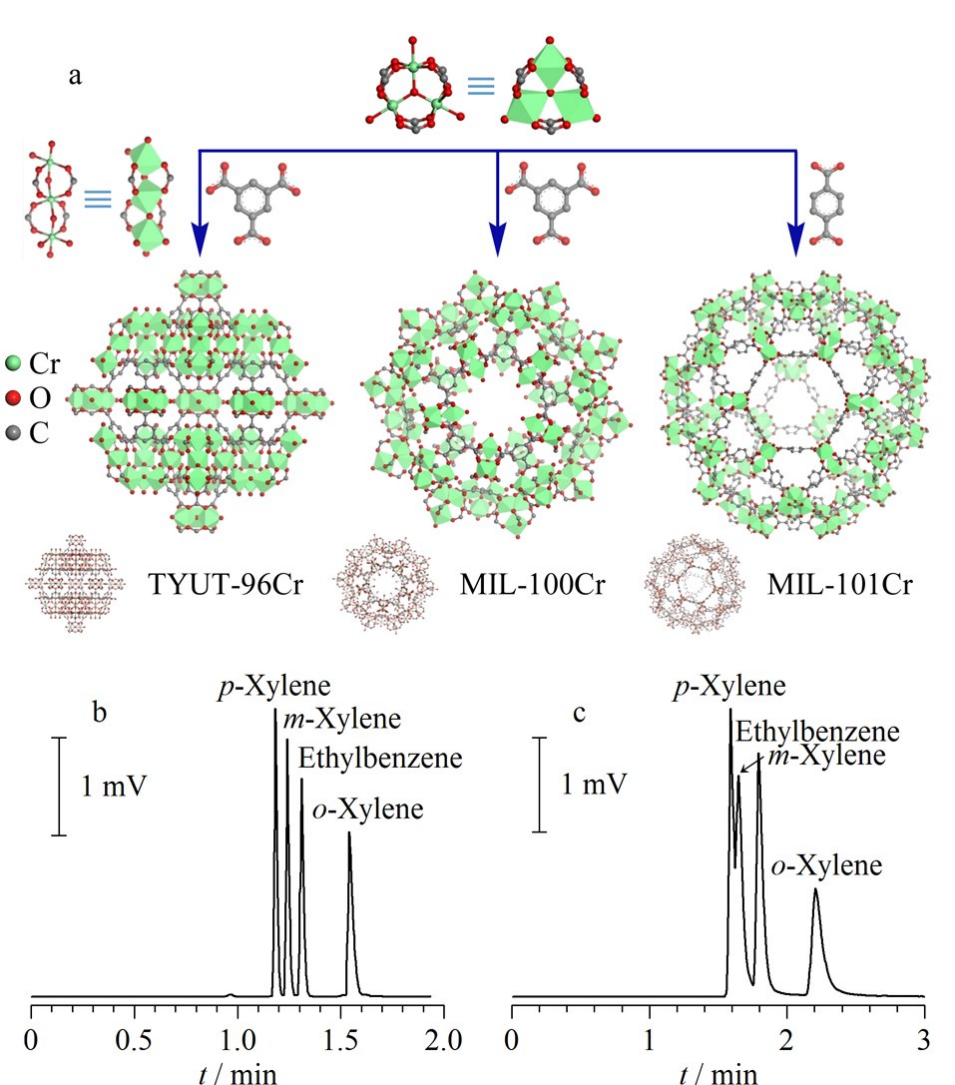
(a)TYUT-96Cr、MIL-100Cr、MIL-101Cr的结构图^[26]^和 (b)MIL-101毛细管柱、(c)吡啶修饰的MIL-101毛细管柱分离二甲苯异构体的气相色谱图^[25]^

Fan等^[27]^以1,3,5-苯三羧酸酯(BTC)为连接体,构建了以氧为中心的金属八面体三聚体(MIL-100(Fe)和MIL-100(Cr))。MIL-100(Fe)和MIL-100(Cr)具有直径为2.5 nm和2.9 nm的两种介孔笼,分析物可从孔径约为0.5 nm和0.9 nm的微孔进入。在没有程序升温的条件下,MIL-100(Fe)毛细管柱可实现烷烃异构体在5 min内的高选择性分离;相同条件下,MIL-100(Cr)毛细管柱不仅没有基线分离烷烃异构体,并且相同分析物的出峰时间约为MIL-100(Fe)毛细管柱的2倍,色谱峰也表现出较严重的拖尾现象。由于这两种MOFs的配体与孔径相同,因此不同的金属中心被认为是造成色谱分离差异的主要原因。与Cr(Ⅲ)相比,Fe(Ⅲ)具有更小的半径,与骨架中氧原子的静电库仑相互作用更强,因此烷烃与MIL-100(Fe)之间的氢键(C-H…O)相互作用更弱。此外,与MIL-100(Cr)相比,MIL-100(Fe)毛细管柱对烷烃表现出更小的焓变与熵变,表明MIL-100(Fe)与烷烃异构体具有更弱的相互作用,烷烃异构体在MIL-100(Fe)中具有更大的自由度。MIL-100(Fe)与MIL-100(Cr)毛细管柱的分离差异表明,通过设计具有适度亲和性的金属中心,可以有效提升气相色谱固定相的分离性能。

除了色谱分离之外,穿透分离的结果也能证实金属亲和性对气体分离的影响。2017年,Yoon等^[28]^合成了MIL-100(M)(M=Al、Cr、Fe),其中MIL-100(Cr)具有最高的不饱和Cr(Ⅲ)位点浓度。在分离N_2_与CH_4_和N_2_与O_2_时,MIL-100(Cr)的不饱和位点能与N_2_分子形成反馈*π*键,表现出较高的N_2_选择性,但MIL-100(Cr)结构中的孔笼直径较大,导致其骨架密度低,单位体积吸附量小。因此Zhang等^[29]^利用Cr与均苯三甲酸进行了空间结构重构,构建了具有优异热稳定性和化学稳定性的微孔TYUT-96Cr。该材料由3种小体积孔笼堆积而成,其中一个孔笼具有一个狭窄的窗口(窗口大小为0.12 nm×0.19 nm),呈现封闭状态,其他两个孔笼由椭圆形窗口(窗口大小为0.72 nm×0.68 nm)相连。活化处理后的TYUT-96Cr具有更高空间密度的Cr活性开放位点,密度泛函理论(DFT)计算结果表明,开放金属位点对N_2_分子显示出更强的亲和力,展现出优异的N_2_吸附体积容量与N_2_分离选择性。TYUT-96Cr对N_2_与O_2_的理想吸附溶液理论(IAST)选择性(10.88)不仅高于传统的商用Li-LSX(8.70)和13X(3.36),并且高于MIL-100Cr(9.10)。随着N_2_浓度减小,TYUT-96Cr的分离性能进一步增强,N_2_与O_2_(体积比为5∶95)的IAST选择性可达26.95,约为MIL-100Cr(15.18)的1.8倍、Li-LSX(10.46)的2.6倍、MIL-101Cr(4.08)的6.6倍、13X(3.24)的8.3倍,在生产高纯氧方面展现出巨大潜力。此外,具有开放金属位点的MOF-74系列材料^[30⇓-32]^、霍夫曼型多孔配位聚合物(M(pz)[Ni(CN)_4_], M=Co、Ni、Fe)^[33]^等MOFs在混合气体分离中也展现出独特的选择性。

### 1.2 *π-π*相互作用

*π-π*相互作用指含有*π*电子体系的分子之间的相互作用。利用多孔MOFs的非共价*π-π*相互作用实现对特定客体分子的高效、选择性分离是促进绿色分离的有效方法。

Tao等^[34]^报道了一种通过简单加热形成的规整堆积的Zr-BTB([Zr_6_O_4_(OH)_4_(BTB)_2_](H_2_O)_4_(OH)_4_(FA)_0.5_)纳米片,其有机配体1,3,5-三(4-羧基苯基)苯(BTB)中包含大量的苯环,能够与不同的芳烃异构体形成*π-π*相互作用,展现出较好的苯系物分离能力。该Zr-BTB色谱柱能够实现对二甲苯异构体、二氯苯异构体、氯甲苯异构体等物质的高效分离。Meng等^[35]^报道了两种纳米级MOFs(NU-1000-N与PCN-608-N),并将两种MOFs动态涂覆至毛细管柱中制得相应色谱柱。NU-1000-N与PCN-608-N色谱柱均展现出良好的取代苯异构体分离能力,这归因于其有机配体1,3,6,8-四(4-羧基苯)芘(H_4_TBApy)与4,4'-二甲氧基联苯-3,3',5,5'-四(4-羧基苯)(L-OMe)中存在的大量苯环,与取代苯异构体之间产生了较强的*π-π*相互作用,为分离创造了一定的有利条件。此外,对NU-1000-N和PCN-608-N的分离效果进行比较,发现NU-1000-N的分离效果更佳。两种材料具有相同的csq拓扑结构,但相对于L-OMe的联苯中心,配体H_4_TBApy的芘中心能够为分析物提供更强的*π-π*相互作用,进而产生了更好的分离效果。因此,通过设计具有合适共轭中心的MOFs固定相,可以分离特定的取代苯异构体。

### 1.3 手性位点

手性化合物广泛存在于自然界中,互为镜像且不可叠加,是生物系统的基本特征。手性化合物的对映异构体通常具有相同的理化性质,但在手性环境中可能存在极大的差异。尤其在手性药物中,两种对映异构体可能表现出不同的药理学和毒理学特征,例如,沙利度胺药物分子的左旋体与右旋体分别具有减轻孕吐反应和致畸性作用^[36]^。因此,手性化合物的分离在众多行业中都极为重要。手性MOFs色谱固定相通常具有高分辨率、高效率、高灵敏度、无液体流动相等优点,主要用于分析热稳定和挥发性外消旋混合物。此外,手性MOFs色谱固定相可以与质谱、固相微萃取(SPME)等技术^[37]^联用,利用手性MOFs对对映体进行气相色谱分离是一种高效的分离方法。

2011年,Xie等^[38]^首次利用手性MOFs([Cu(sala)]*_n_*)作为气相色谱固定相,进行高分辨率的外消旋体分离。[Cu(sala)]*_n_*是一种单手性螺旋配位聚合物,在加热过程中单链螺旋通过失去水分子并交联产生手性开放通道。他们利用动态涂覆法制备了[Cu(sala)]*_n_*毛细管柱,凭借其优异的热稳定性和三维手性通道,成功分离了香茅醛、樟脑、丙氨酸、1-苯基乙醇等11种对映异构体,展示出优异的对映选择性和分离能力。此外,一系列具有螺旋结构的手性MOFs毛细管柱([Cd(LTP)_2_ ${]}_{n}^{\left[39\right]}$ 、Co(D-Cam)_1/2_(bdc)_1/2_(tmdpy)^[40]^、InH(D-C_10_H_14_O_4_ ${)}_{2}^{\left[41\right]}$ )被成功应用于外消旋体的分离,且具有更好的对映异构体分离能力。

2017年,Li等^[42]^报道了一种手性MOFs(Co-L-GG),该材料具有较高的比表面积和稳定的理化性质。研究发现,该手性柱能够分离30种有机化合物的外消旋体,与其他手性柱相比,该手性柱具有广泛的手性识别和对映异构体选择性分离能力(图3)。2018年,Kou等^[43]^通过将具有各种识别位点的手性基团修饰到铝基MOFs(MIL-101-NH_2_)中,获得了5种手性MOFs,经过动态涂覆制得相应毛细管柱。与商用手性毛细管柱相比,该手性MOFs毛细管柱具有更优异的对映体分离性能。由此可见,通过设计不同的手性位点能够在MOFs中创造不同的手性环境,以实现不同分析物的高效分离。

**图 3:**
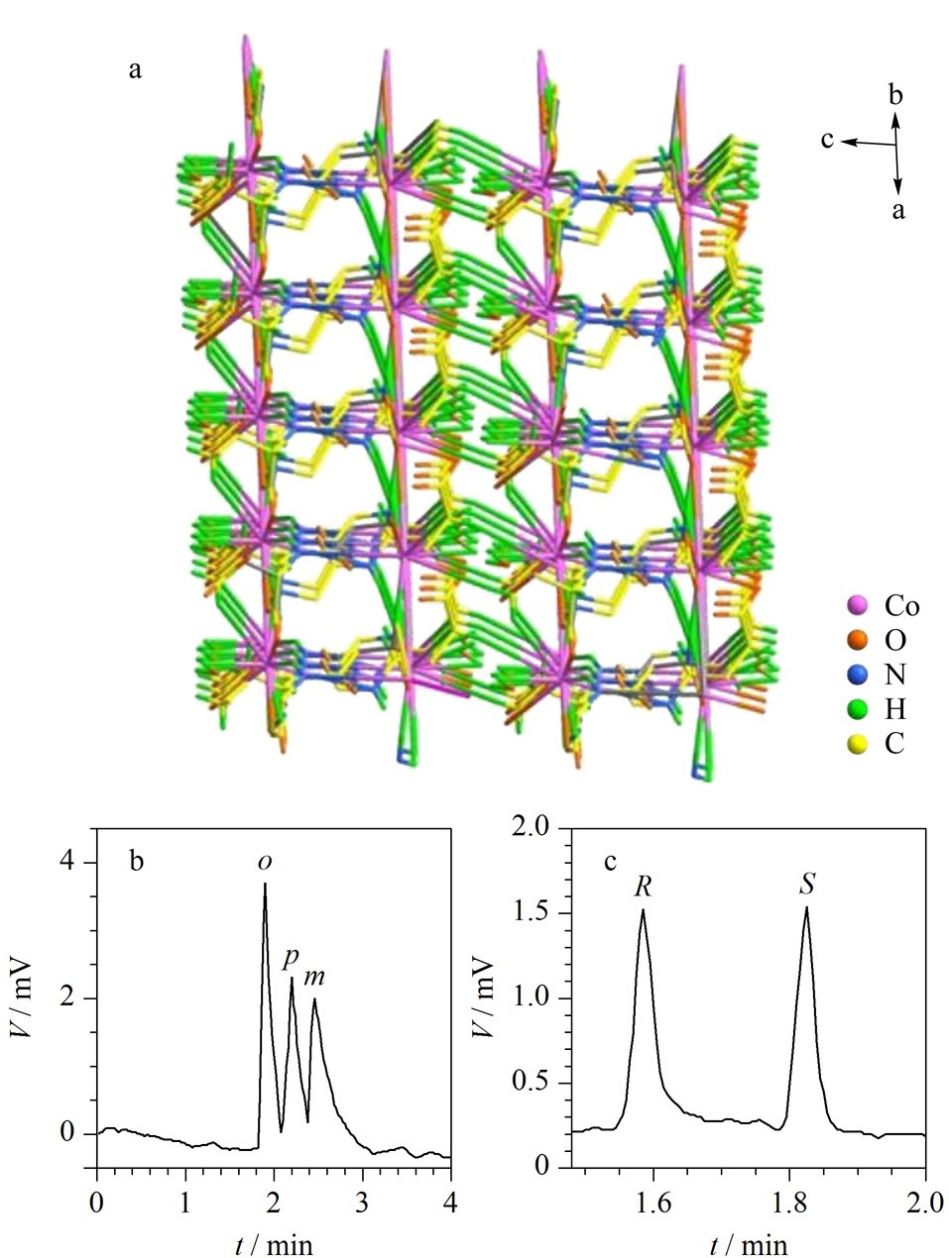
(a)手性MOF(Co-L-GG)的三维结构图和Co-L-GG 手性柱分离(b)硝基甲苯异构体、(c)2-乙基己酸的气相色谱图^[42]^

### 1.4 极性

在MOFs合成过程中加入极性基团能够改变其表面性质和化学性质^[44,45]^,进而对色谱分离效果产生影响。极性基团可以使色谱柱具有更强的极性,与具有相似结构或化学基团的分析物之间产生更强的相互作用,同时MOFs表面的孔隙率、孔径尺寸及形状可能会因极性基团的加入而发生变化,从而改变其分离选择性。

Gu等^[46]^利用脉冲气相色谱、静态气相吸附和突破吸附等方法,研究了两种锌基MOFs(MOF-5和单斜MOF)对二甲苯异构体的吸附分离。两种MOFs柱的极性不同,分别表现出不同的选择性和分离效率。在MOF-5柱中,乙基苯最早被洗脱,其他3种二甲苯异构体被同时洗脱,而单斜MOF色谱柱具有对二甲苯选择性,无法分离二甲苯异构体与乙基苯。根据测得的麦氏常数,MOF-5柱的5项麦氏常数平均值甚至小于非极性角鲨烷,因此属于非极性色谱柱。单斜MOF色谱柱中苯、硝基丙烷和吡啶的麦氏常数较大,其极性强于MOF-5柱,因此Gu等^[46]^认为非极性相有利于二甲苯异构体与乙基苯的热力学分离。Meng等^[35]^将具有不同配体、相同拓扑的两种锆基MOFs(NU-1000(Zr_6_O_4_(OH)_8_(H_2_O)_4_(TBAPy)_2_)和PCN-222(Zr_6_(μ_3_-O)_4_(μ_3_-OH)_4_(OH)_4_(H_2_O)_4_(FeTCPPCl)_2_))制备成毛细管色谱柱,并对比了这两种色谱柱的分离性能(图4)。在分离取代苯异构体时,NU-1000的分离性能远优于PCN-222,这可能是由于PCN-222的卟啉配体提供了过强的氢键,不利于取代苯异构体的脱附。

**图 4:**
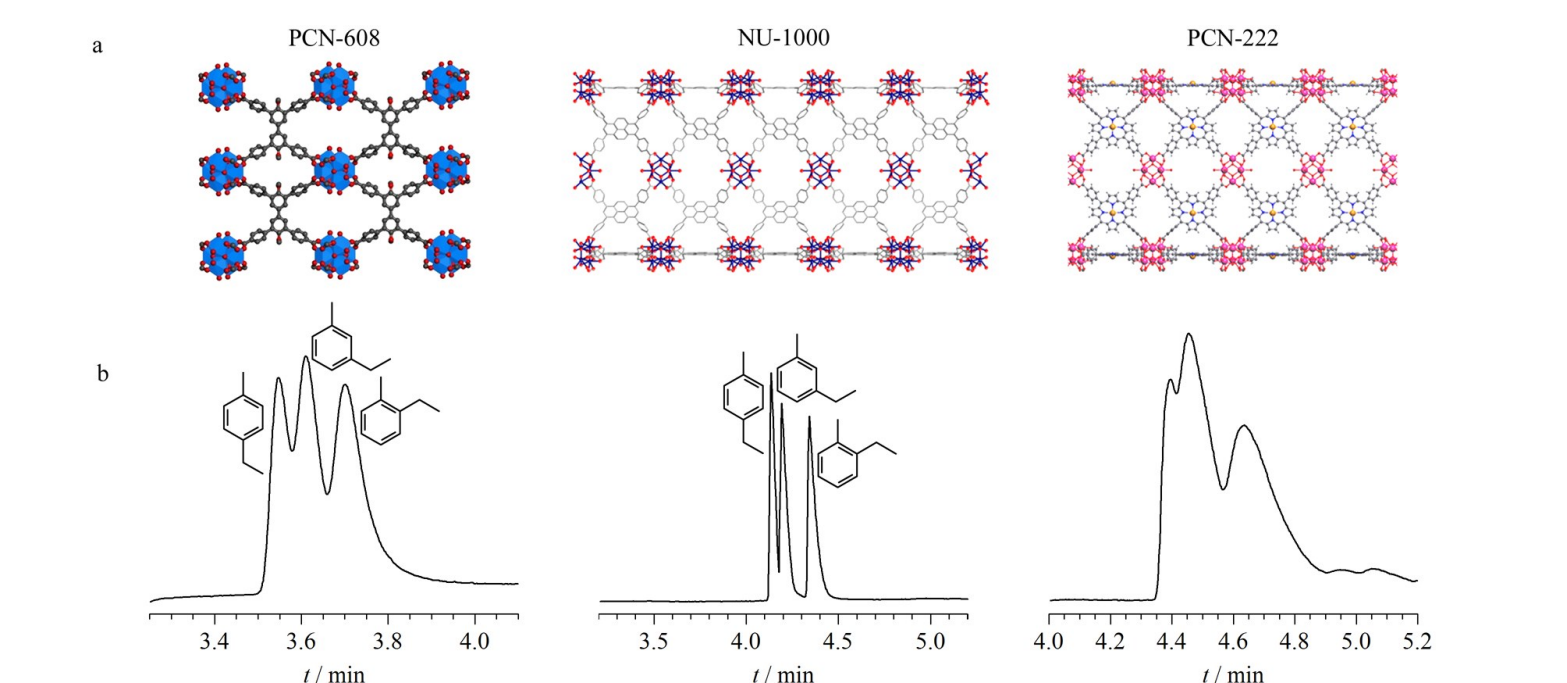
(a)PCN-608、NU-1000、PCN-222的结构图^[47⇓-49]^和(b)PCN-608-N、NU-1000-N、PCN-222-N毛细管柱分离乙基甲苯异构体的气相色谱图^[35]^

类似地,在穿透分离小分子的过程中,MOFs的极性也会影响其分离性能。如Gu等^[50]^通过氨基功能化,将路易斯碱性位点固定到高度稳定且具有可调性的锆基MOFs(UiO-67)^[48,49]^中,获得同样具有fcu拓扑结构的UiO-67-(NH_2_)_2_,用于乙炔、乙烯、乙烷的分离;其中,引入的氨基明显改变了UiO-67的孔隙特性,其八面体笼的孔径由1.75 nm减小到1.15 nm,四面体笼被分割为0.71 nm和0.52 nm的两种笼状口袋,为分析物提供了合适的孔径限制。与UiO-67相比,氨基的引入提供了额外的吸附位点,乙炔分子与3个氨基结合形成6个氢键(3个C-H…N和3个N-H…C)相互作用,乙烷分子与3个氨基结合形成3个C-H…N相互作用,相比之下,乙烯分子仅增加了两个C-H…N相互作用。因此,乙炔、乙烷与UiO-67-(NH_2_)_2_的结合能力比乙烯更强。UiO-67-(NH_2_)_2_实现了乙炔、乙烷的选择性吸附,并表现出环境条件下目前最高的乙炔和乙烷吸附量,同时实现了C_2_三元混合物中乙烯的一步高效分离,为高纯乙烯高效、节能一步分离提供了新思路。

## 2 动力学扩散

动力学扩散是指气体分子在孔隙内的非障碍性扩散,在色谱分离过程中起着非常重要的作用。在宏观尺度上,动力学因素通常影响色谱峰形与色谱柱效能。气相色谱动力学统一方程中的*μ*_3_项通常被用来描述色谱峰的峰形:

*μ*_3_=*μ*_3,_*_j_*+ $\frac{12LD_{m}^{2}}{u^{5}}$ (1+*k*)^3^+ $\frac{12LD_{m}}{u^{3}}$ *k*(1*+k*)

$\left(\frac{k}{k_{m}\xi }+\frac{d_{f}^{2}}{D_{s}}\right)$ + $\frac{6L}{u}$ k ${\left(\frac{k}{k_{m}\xi }+\frac{d_{f}^{2}}{D_{s}}\right)}^{2}$

式中*u*为线流速(m/s), *d*_f_为固定相厚度(m), *L*为柱长(m), *D*_m_为流动相中分析物的分子扩散系数(m^2^/s), *k*_m_*ξ*为气相传质系数,*D*_s_为固定相中分析物的分子扩散系数(m^2^/s); *D*_s_值越大表示分析物在孔内的扩散速率越大,相应的色谱峰越尖锐、对称,反之则会出现较宽的拖尾峰。此外,色谱柱效能可以根据戈雷方程进行评价:

*H*= $\frac{2D_{g}}{u}$ +

$\left[\frac{1+6k+11k^{2}}{24{\left(1+k\right)}^{2}}\times \frac{r^{2}}{D_{g}}+\frac{2}{3}\times \frac{k}{{\left(1+k\right)}^{2}}\times \frac{d_{f}^{2}}{D_{s}}\right]$ ×*u*

式中*H*为理论塔板高度(m), *D*_g_为分析物在气相中的分子扩散系数(m^2^/s), *r*为毛细管半径(m)。该方程可简写为*H=B/u*+*Cu*,其中*D*_s_所在项(*Cu*)为传质阻力项(m),分析物在固定相中的传质阻力随扩散系数增大而减小。传质阻力的减小会导致色谱柱的理论塔板高度减小、理论塔板数增加,色谱柱表现出较高的柱效。

固定相的孔径大小对客体分子的动力学扩散速率具有较大的影响。较大的孔径使得客体分子在孔隙内移动时受到较小的阻力,从而能够快速通过孔道,而小孔径的固定相具有分子筛分作用,能有效提升气体混合物的分离选择性。此外,固定相的颗粒尺寸与堆积模式同样也会影响动力学扩散和气体分离效率。因此,从适当提高客体分子的动力学扩散角度对MOFs的结构进行调整可以进一步提高色谱分离效率。

### 2.1 体积排阻

设计和修饰MOFs的孔道形状及尺寸对于提高材料的分离性能起着至关重要的作用^[51,52]^。通过设计具有合适孔径的固定相,能够有效阻止体积较大的分析物进入孔道,而与孔径相匹配的较小分子可以顺利进入孔道,进而表现出较长的保留时间。

2010年,Chang等^[53]^首次制备了具有MOFs尺寸排阻效应的沸石-咪唑框架材料(ZIF-8)毛细管色谱柱。与MOF-508^[54]^和MOF-5^[46]^毛细管柱的沸点出峰顺序不同,ZIF-8毛细管柱中支链烷烃的出峰时间远小于直链烷烃,展现出独特的烷烃分离选择性和较高的分离度(图5)。这归因于ZIF-8狭窄的孔径仅允许直链烷烃进入孔腔,与内壁产生较强的范德华力相互作用。因此,直链烷烃能够得到较好的分离,而体积较大的支链烷烃无法进入,仅与孔腔外壁产生较弱的范德华力相互作用,难以被分离。此外,该毛细管柱还能根据沸点对直链烷烃进行高分辨率气相色谱分离。随着直链烷烃的长度不断增加,其与孔腔内壁的范德华力相互作用会不断增强,表现出更长的保留时间。

**图 5:**
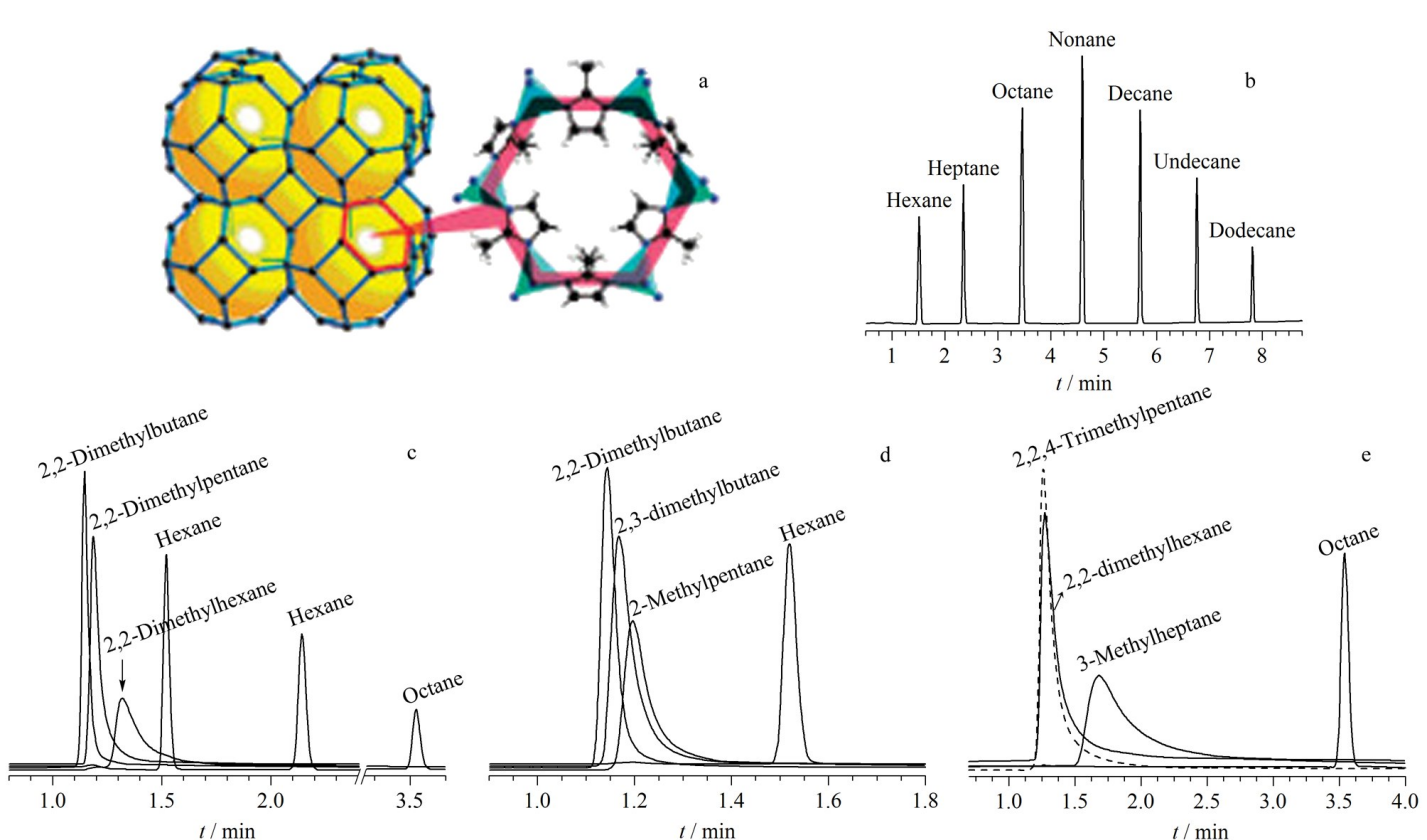
(a)ZIF-8的结构图和ZIF-8毛细管柱分离(b)直链烷烃、(c)2,2-二甲基支链烷烃和直链烷烃、(d)己烷及其支链异构体、(e)辛烷及其支链异构体的气相色谱图^[53]^

Meng等^[55]^通过变换金属中心、改变配体长度对含有 ${{\mathrm{SiF}}_{6}}^{2-}$ 的微孔材料(SIFSIX-1-Zn)进行了精确地孔径调控,获得了具有互穿结构的材料SIFSIX-1-Cu^[56]^和超微孔材料SIFSIX-3-Zn(图6a),并将其制备成毛细管色谱柱用于烷烃的分离。动力学直径为0.43 nm的线性烷烃与SIFSIX-3-Zn中0.38 nm的孔径相匹配,能与具有静电性的 ${{\mathrm{SiF}}_{6}}^{2-}$ 产生较强的氢键(C-H…F)相互作用,因此,SIFSIX-3-Zn对直链烷烃和支链烷烃展现出良好的基线分离能力。孔径为0.83 nm的SIFSIX-1-Zn仅能提供较弱的相互作用,而SIFSIX-1-Cu的互穿结构导致其孔径小于线性烷烃的临界尺寸,因而两者对烷烃的分离选择性远不如具有尺寸排阻效应的SIFSIX-3-Zn(图6b)。三者的分离差异表明,改变配体长度以设计具有合适孔径的MOFs固定相能有效提升分析物的分离效率。

**图 6:**
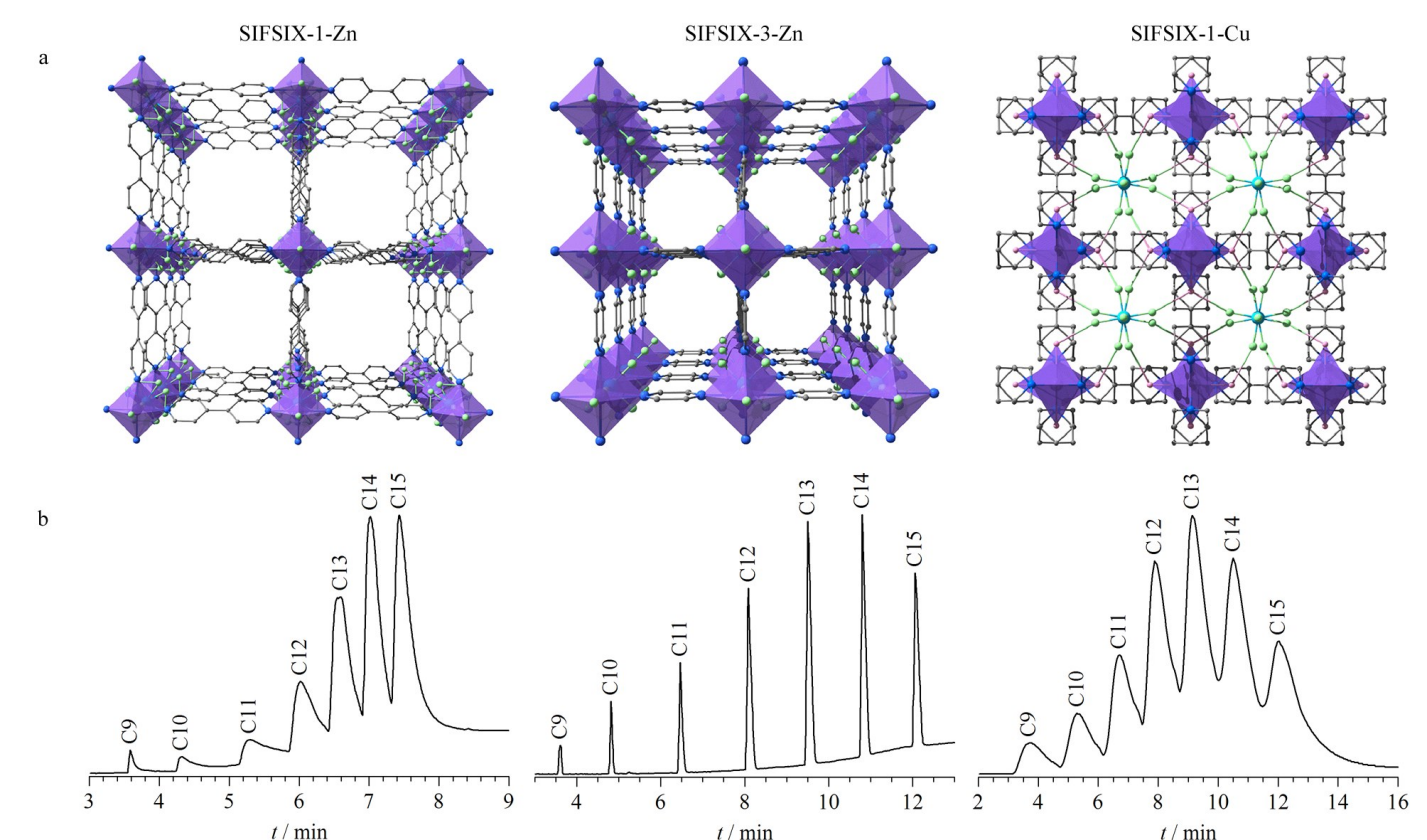
(a)SIFSIX-1-Zn、SIFSIX-3-Zn和SIFSIX-1-Cu沿c轴的结构图以及(b)SIFSIX-1-Zn、SIFSIX-3-Zn和SIFSIX-1-Cu毛细管柱对直链烷烃的气相色谱分离图^[55]^

### 2.2 颗粒尺寸

根据范蒂姆特方程,增强分析物的动力学扩散能够改善MOFs固定相的分离效果。除了改变MOFs的自身结构外,可以通过减小MOFs固定相的粒径来改善分析物的扩散速率^[57]^。一方面减小粒径可以产生更为规整的堆积孔道,另一方面可以缩短扩散路径,这些都能有效降低分析物所受到的扩散阻力。较低的反应物浓度、过量的配体(或调节剂)、强金属-配体键与低质子活性是形成纳米级MOFs的理想条件^[58]^。

NU-1000^[59,60]^、PCN-608^[9]^、PCN-222^[61]^是具有相同csq拓扑结构的3种锆基MOFs,分别由3种不同的配体与锆簇相连,展现出不同的分离性能。Meng等^[35]^在微米级尺寸的基础上,分别利用调节水量、缩短反应时间等调控方式合成了纳米级NU-1000-N、PCN-608-N(图7a)、PCN-222-N。与相对应的微米级相比,其结构相同但粒径不同。在烷烃和取代苯异构体的分离中,纳米级NU-1000-N、PCN-608-N和PCN-222-N涂覆的毛细管色谱柱表现出比其相应的微米级MOFs更高的分离性能,且峰展宽和峰拖尾现象明显减少(图7b)。以相同的分析物研究扩散阻力,结果表明,140 ℃下分析物在纳米级NU-1000-N中的传质阻力系数(*C*)为0.0048 s,在微米级NU-1000-M中的*C*为0.1815 s。因此,减小MOFs的粒径可以缩短分析物的扩散路径,削弱传质阻力,使分析物在MOFs中的动力学扩散得到增强,以实现高效色谱分离。

**图 7:**
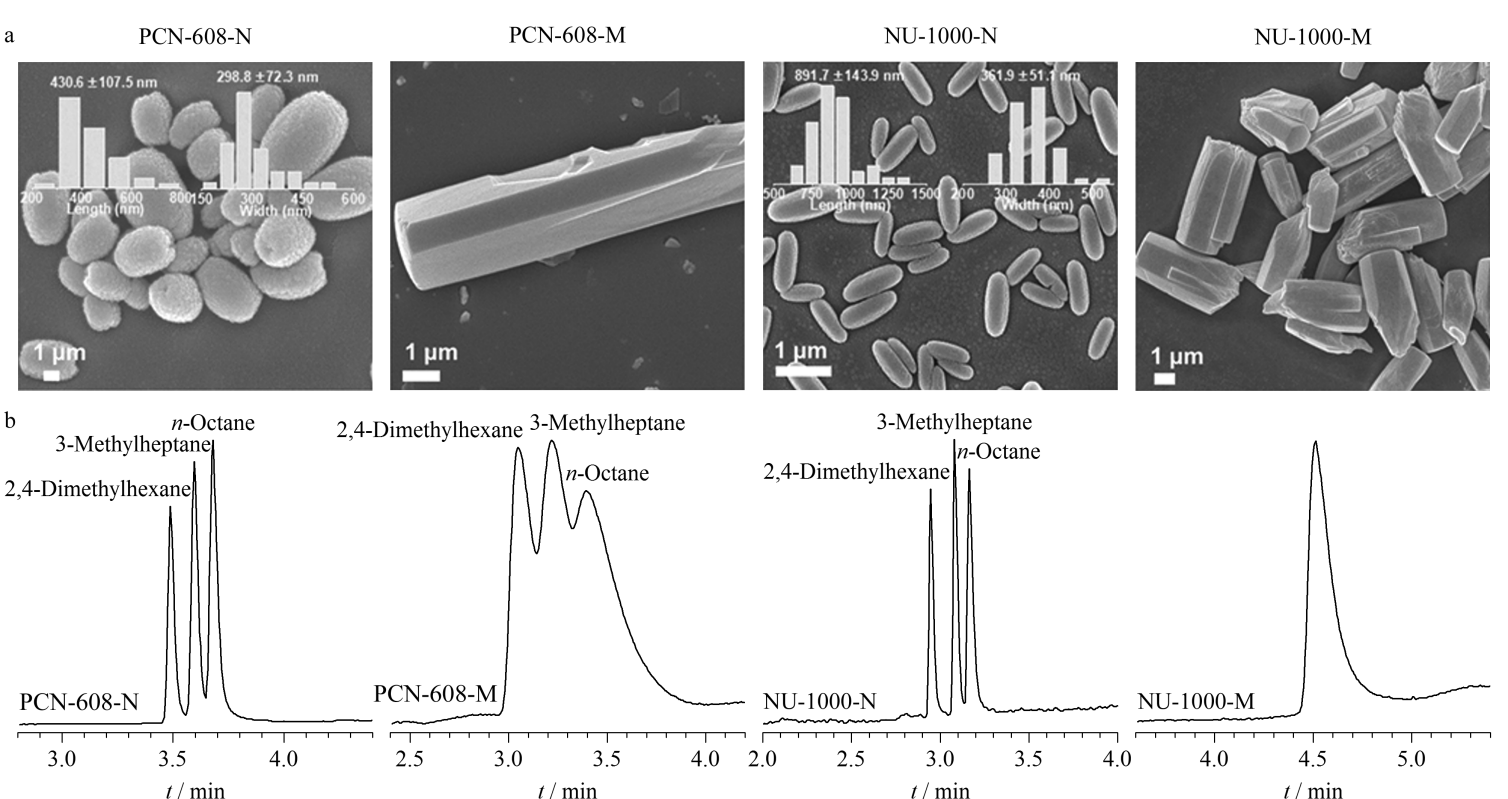
(a)纳米级与微米级PCN-608、NU-1000的扫描电子显微镜(SEM)图像和(b)纳米级与微米级PCN-608、NU-1000毛细管柱对辛烷及其支链异构体的气相色谱分离对比图^[35]^

Xu等^[62]^将3种纳米级NU-901动态涂覆至毛细管色谱柱中,小粒径的纳米级NU-901降低了颗粒内部的分子扩散势垒,同时均匀的涂层有效降低了扩散阻力,使理论塔板数(*N*)增加(*N*_NU-901-NP_=1127 plates/m,*N*_bulk NU-901_=419 plates/m)。因此,纳米级NU-901表现出更好的色谱分离能力。此外,一些纳米级MOFs,如IRMOF-1、IRMOF-3^[63]^也被报道作为气相色谱固定相用于混合气体的分离。

### 2.3 堆积模式

二维MOF纳米片是一种超薄、具有多孔特性的新型材料。与三维材料相比,二维MOF纳米片更有利于分析物的扩散,但在合成过程中会不可避免地形成纳米片的随机堆叠,导致其分离性能降低。由于纳米片的组成一致,难以从热力学作用力角度改善其分离性能,但通过从动力学角度调控堆积方式可以调控其孔径大小和分子扩散路径、速率等,从而进一步影响其分离性能。

Tao等^[34]^利用自下而上法合成了二维纳米片Zr-BTB,并且通过调控纳米片的堆积结构以形成规整堆叠,实现对气体分子的高度选择性分离。在高角环形暗场扫描透射(HAADF-STEM)图像中观察到,未经特殊处理的纳米片呈现8°、14°和30° 3个特定角度为主的随机堆叠。将随机堆叠的纳米片在乙醇中浸泡后进行真空加热处理发现,随机堆叠的纳米片变为规整堆叠,这是由于在加热过程中Zr-BTB纳米片相邻层间的乙氧基基团不断减少,层间的Zr簇以Zr-O-Zr键的形式相连,产生了较强的相互作用,形成了高度有序的亚纳米孔。随机堆叠的Zr-BTB纳米片对二甲苯异构体等苯系物几乎没有分离能力,而规整堆积的Zr-BTB纳米片具有良好的分离性能,其成功分离了二甲苯、氯甲苯、二氯苯等6组异构体,并展现出独特的对位选择性(图8a)。与传统的扭曲堆积方式不同,这种规整的堆积方式可以提高二维MOF纳米片的孔隙率和分子扩散速率,从而实现更高选择性的吸附和分离。

**图 8:**
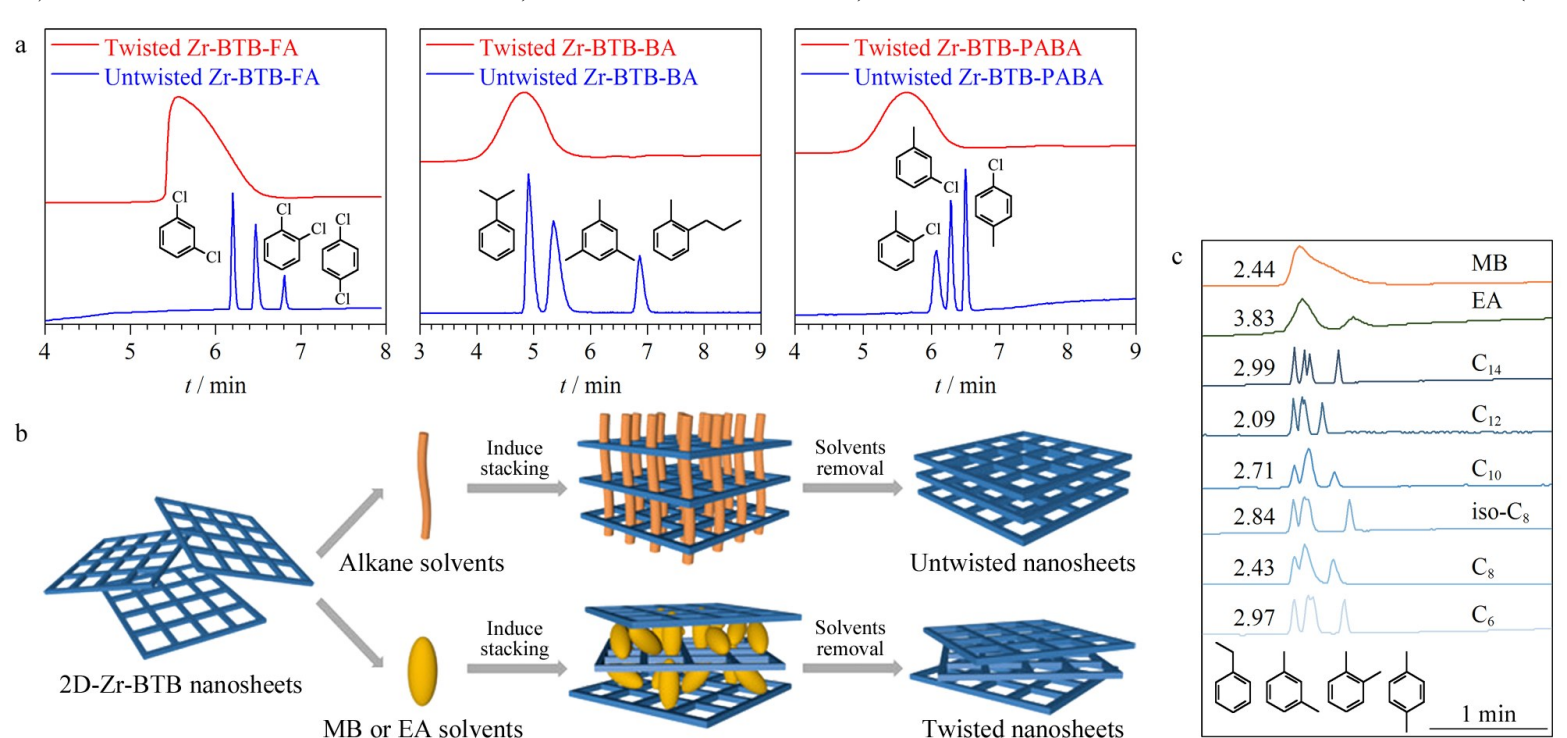
(a)扭曲堆积与规整堆积的Zr-BTB毛细管柱对苯系物的气相色谱分离图^[34]^, (b)利用非共价相互作用调控2D-Zr-BTB堆积模式的示意图^[64]^以及(c)不同溶剂洗涤的2D-Zr-BTB对二甲苯异构体的气相色谱分离图^[64]^

Tang等^[64]^利用不同的溶剂分子诱导主客体产生非共价相互作用(图8b),从分子水平上进一步探究了二维金属有机骨架纳米片的堆积模式对纳米片的性能与应用的影响。甲基苯(MA)与乙酸乙酯(EA)诱导的Zr-BTB纳米片扭曲堆积分别表现出12°、18°、24°和6°、18°、24°、30°的旋转角;而正己烷、正辛烷等具有疏水性的非极性烷烃倾向于垂直进入Zr-BTB纳米片的孔道,诱导纳米片由扭曲堆积转变为规整堆积。两种堆积模式分别产生了不同尺寸的孔隙,扭曲堆积的孔径大小约为0.59 nm和0.66 nm,规整堆积的孔径约为0.59 nm。不同堆积方式的Zr-BTB纳米片展现出对苯衍生物异构体不同的分离性能(图8c),其中规整堆积的纳米片表现出优于扭曲堆积纳米片的分离效果,成功基线分离了二甲苯和乙基苯、三氯苯和氯甲苯等异构体,同时展现出独特的对位选择性。与传统的共价键连接方式不同,这种非共价相互作用可以实现对纳米片的精确控制和调控,从而形成高度有序的堆积结构,提高异构体的色谱分离效率。

Xu等^[62]^通过调节水含量和不同单体酸的浓度,自下而上合成了不同形态的纳米级NU-901(超薄纳米片NU-901-NS、互穿纳米片NU-901-I-NS和纳米颗粒NU-901-NP),在溶剂去除过程中,3种材料分别表现出规整堆积、随机堆积和紧密堆积。不同堆积模式的NU-901产生了不同的孔隙环境,分别展现出对氯甲苯、乙基甲苯等异构体不同的色谱分离能力。规整堆积的NU-901-NS产生了规则的微孔(约0.6 nm),具有一定的色谱分离能力,而NU-901-I-NS的随机堆积降低了其孔隙率和孔隙体积,阻碍了分析物的扩散,导致色谱分离能力较差;紧密堆积的NU-901-NP颗粒间孔隙小,孔径分布最均匀,在3种材料中表现出最好的分离能力。与按照分析物沸点顺序出峰的HP-5MS商业柱相比,3种纳米级NU-901展现出独特的对位选择性,这源于对位异构体与孔径间匹配的尺寸,使对位异构体与MOFs固定相能够产生更强的相互作用,表现出最长的保留时间。因此,MOFs的堆积调控对气体分离有着重要的影响,选择适当的堆积模式可以调节MOFs的孔道、分子扩散速率和稳定性,从而实现对不同气体的高效分离。

## 3 热力学相互作用与动力学扩散的协同效应

在分离过程中,分析物与固定相间的热力学相互作用和分析物的动力学扩散之间存在一种平衡状态,如果只考虑热力学相互作用,则需要较多的作用位点来捕获目标分子,但这可能会导致分析物在固定相中具有较低的扩散速率,产生严重的拖尾现象;相反,如果只考虑分析物的扩散,那么MOFs应该具有允许更多分子通过的最佳孔径结构,但这会导致分析物与固定相间的相互作用不足,表现为较短的保留时间和较差的分离效果。因此,通过合理设计多孔材料的结构来平衡分析物与固定相间的热力学相互作用以及分析物的动力学扩散,能够有效提高MOFs材料的分离性能^[65⇓⇓⇓-69]^。

Xu等^[70]^首次提出MOFs固溶体(MOSS)的合成策略,通过半配体诱导混合NU-901(微孔)与NU-1000(介孔)生成均匀的MOSS单纳米颗粒。随着半配体(L_B_)不断增加,MOSS的介孔比例呈现出先增加后减少的趋势,将其涂覆于毛细管气相色谱柱中,MOSS展现出良好的二取代苯和烷烃异构体分离性能(图9a)。此外在分离二甲苯异构体时,二甲苯异构体在MOSS涂覆的色谱柱上展现出了独特的出峰顺序,这是由于MOSS的混合微介孔结构较好地平衡了分析物的动力学扩散与热力学相互作用。在具有纯微孔的NU-901中,尺寸较小的对二甲苯更易进入,并与孔壁发生较强的相互作用,从而具有更长的保留时间;在具有大量介孔的NU-1000中,对二甲苯与孔壁的作用力最小,从而表现出最短的保留时间;其中微介孔比例为1∶1的MOSS-3具有最优的分离性能,这是由于优化的微介孔比例平衡了气体在MOFs中的热力学相互作用与动力学扩散,体现出更高的分离分辨率与独特的洗脱顺序。

**图 9:**
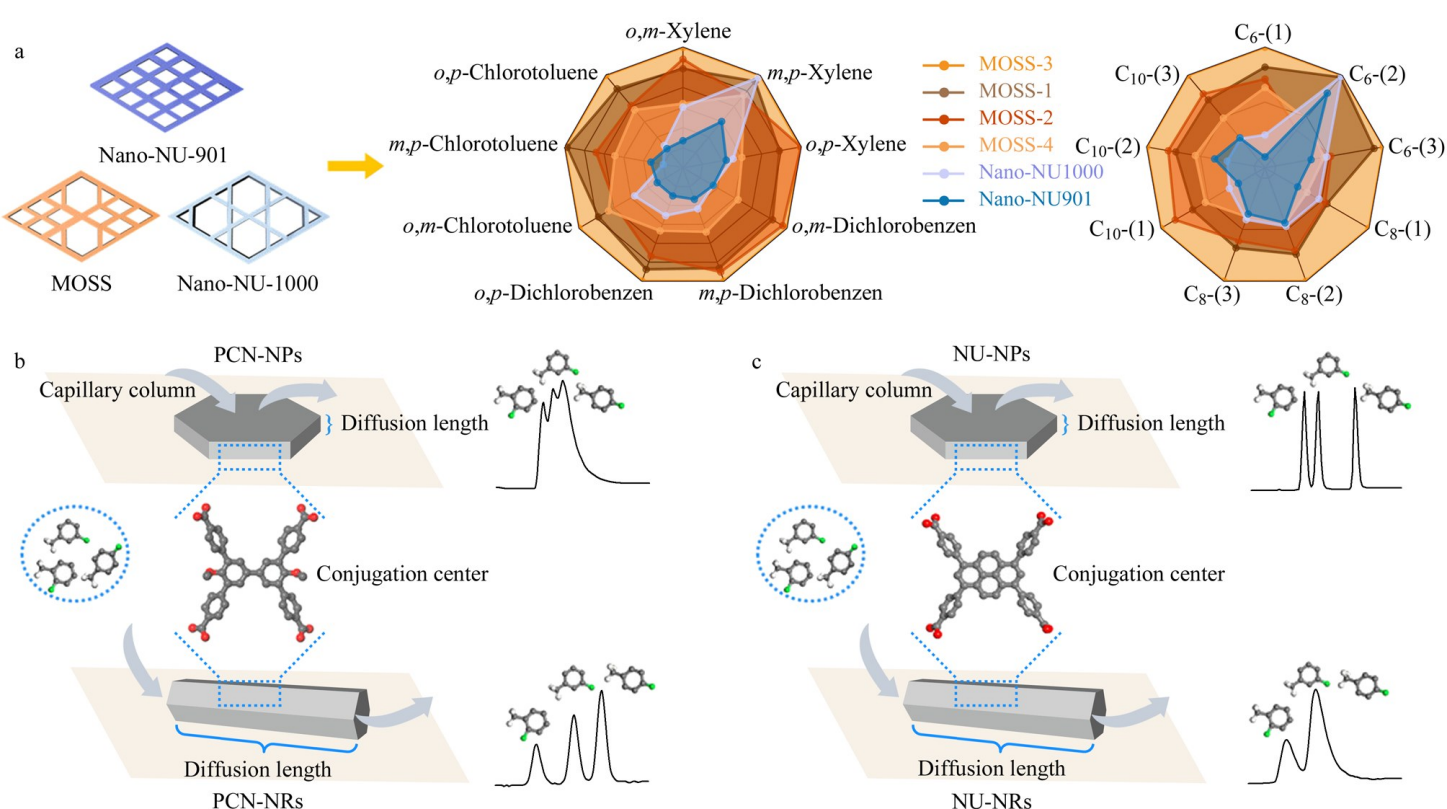
(a)二取代苯异构体与烷烃异构体在NU-901、NU-1000、MOSS毛细管柱上的分离性能^[70]^和扩散路径与作用中心对(b)PCN材料和(c)NU材料分离性能的影响示意图^[47]^

最近该团队利用配体剪裁策略,根据PCN-608原有配体4,4'-二甲氧基联苯-3,3',5,5'-四(4-羧基苯)(L-OMe)的晶面配位结构和形状,沿着长轴与短轴将其“裁剪”成L_B_-1、L_B_-2两种不同形状的封端剂,以控制PCN-608特定晶面的生长速度,进而获得具有不同形貌的PCN-608材料^[47]^。其中L_B_-1仅能配位到{001}晶面,在生长过程中减缓了晶体中{001}晶面的生长速度,缩短了PCN-608的长径比,且随着L_B_-1不断增加,能够获得不同长径比的六棱台(PCN-NPs)。同样地,L_B_-2的加入减缓了晶体中{100}晶面的生长速度,进而延长了晶体的长径比,获得了具有不同长径比的六棱棒(PCN-NRs)。同时利用NU-MOFs验证了该策略的普适性,结果表明,随着封端剂L_B_-1或L_B_-2的不断加入,同样可以获得具有不同长径比的NU-MOFs。在利用气相色谱探索其孔道差异时发现,在PCN-MOFs中,扩散路径较长的PCN-NRs具有更好的分离性能;而在NU-MOFs中,扩散路径较短的NU-NPs更有利于分析物的分离。这是由于PCN-MOFs(PCN-NPs和PCN-NRs)的联苯中心所提供的热力学相互作用较弱,在延长扩散路径后,分析物与联苯中心作用的次数增加,有利于平衡固定相与分析物之间的热力学相互作用和分析物的动力学扩散,从而展现出更好的分离性能;而NU-MOFs具有较大的共轭中心,能够提供过强的作用力,在缩短扩散路径后,分析物的扩散阻力减小,更有利于平衡固定相与分析物之间的热力学相互作用和分析物的动力学扩散,提高材料的分离性能(图9b、c)。

## 4 总结与展望

组成MOFs的金属中心和配体十分多样,如何选择合适的金属盐、有机配体与调节剂,合成具有高比表面积、高稳定性的MOFs固定相以分离特定目标物,尚未形成明确的设计思路。在此,我们总结了从热力学相互作用与动力学扩散角度设计高效MOFs固定相的设计思路。在热力学相互作用层面,可以通过在MOFs中设计合适的热力学作用位点实现分析物的高效分离,如通过选择含芳香结构的有机配体与分析物产生*π-π*相互作用,添加不同的手性位点以形成不同的手性环境,选择不同的金属中心与分析物产生不同的亲和性,以及添加不同的极性基团以产生不同的极性环境等。在动力学层面,可以从提高客体分子动力学扩散速率的角度出发,通过选择不同尺寸的配体以调节MOFs的孔径尺寸、粒径与堆积等方式以实现高效色谱分离。此外,我们提出了可以从平衡客体分子与固定相之间的热力学相互作用以及客体分子的动力学扩散角度出发,合理设计多孔材料的结构以实现高效的分离性能,为高性能固定相的设计提供了思路。

现有MOFs色谱柱已成功应用于气体的吸附分离,但这些色谱柱仍存在一定的局限性。如在分离烯烃、烷烃、氢气、氮气等小分子气体时,商业化色谱柱可以实现小分子气体的快速高效分离,而MOFs色谱柱存在保留较弱、分离选择性低、柱流失等问题,因此如何提高其长期稳定性与分离性能是一项有待进一步研究的课题。此外,在未来可以进一步利用计算机模拟等技术来设计多孔材料,以实现更高效、精准的气体分离和分子筛选,如利用分子动力学模拟、密度泛函理论等方法来预测MOFs的结构与性质,进一步指导实验设计。总之,希望我们的综述能够为固定相的设计提供一定的思维启发。

## References

[1] Sholl D, Lively R. Nature, 2016, 532: 435 27121824 10.1038/532435a

[2] Yang Y X, Bai P, Guo X H. Ind Eng Chem Res, 2017, 56(50): 14725

[3] Ambrose D. Nature, 1964, 202: 116

[4] Zhang S N, Zheng Y L, An H D, et al. Angew Chem Int Ed, 2018, 57(51): 16754 10.1002/anie.201810571 30359485

[5] Lee Y R, Do X H, Cho K Y, et al. ACS Appl Nano Mater, 2020, 3(10): 9852

[6] Chen X X, Liu M Y, Zhang L J, et al. Int J Hydrogen Energy, 2021, 46(24): 13029

[7] Yui Y, Miyazaki S, Ma Y, et al. J Chromatogr A, 2016, 1450: 45 27157422 10.1016/j.chroma.2016.04.076

[8] Lin Y F, Wan H, Wu D, et al. J Am Chem Soc, 2020, 142(16): 7317 32248690 10.1021/jacs.0c01916

[9] Pang J D, Yuan S, Qin J S, et al. J Am Chem Soc, 2019, 141(7): 3129 30689379 10.1021/jacs.8b12530

[10] Usman M, Helal A, Abdelnaby M M, et al. Chem Rec, 2021, 21(7): 1771 10.1002/tcr.202100030 33955166

[11] Polyukhov D M, Poryvaev A S, Sukhikh A S, et al. ACS Appl Mater Interfaces, 2021, 13(34): 40830 34423631 10.1021/acsami.1c12166

[12] Zheng H L, Huang S L, Luo M B, et al. Angew Chem Int Ed, 2020, 59(52): 23588 10.1002/anie.202012019 32926488

[13] Finsy V, Verelst H, Alaerts L, et al. J Am Chem Soc, 2008, 130(22): 7110 18470988 10.1021/ja800686c

[14] Alaerts L, Maes M, Giebeler L, et al. J Am Chem Soc, 2008, 130(43): 14170 18826226 10.1021/ja802761z

[15] Jia T, Gu Y F, Li F T. J Environ Chem Eng, 2022, 10(5): 108300

[16] Lin R B, Xiang S C, Zhou W, et al. Chem, 2020, 6(2): 337

[17] Zhao X, Wang Y, Li D S, et al. Adv Mater, 2018, 30(37): e1705189 29582482 10.1002/adma.201705189

[18] Li H, Li L B, Lin R B, et al. Energy Chem, 2019, 1(1): 100006

[19] Idrees K B, Li Z, Xie H M, et al. J Am Chem Soc, 2022, 144(27): 12212 35786875 10.1021/jacs.2c03114

[20] Tang W Q, Meng S S, Xu M, et al. Chinese Journal of Chromatography, 2021, 39(1): 57 34227359 10.3724/SP.J.1123.2020.06028 PMC9274853

[21] Rajkó R, Körtvélyesi T, Sebök Nagy K, et al. Anal Chim Acta, 2005, 554(1/2): 163

[22] You W Q, Liu Y, Howe J D, et al. J Phys Chem C, 2018, 122(48): 27486

[23] Ha H, Kim Y, Kim D, et al. Chem Eur J, 2019, 25(63): 14414 10.1002/chem.201903210 31441970

[24] Zhai Q G, Bu X H, Mao C Y, et al. J Am Chem Soc, 2016, 138(8): 2524 26894827 10.1021/jacs.5b13491

[25] Gu Z Y, Yan X P. Angew Chem Int Ed, 2010, 49(8): 1477 10.1002/anie.200906560 20091724

[26] Zhang F F, Li K J, Chen J, et al. Sep Purif, 2022, 281: 119951

[27] Fan L, Yan X P. Talanta, 2012, 99: 944 22967647 10.1016/j.talanta.2012.07.063

[28] Yoon J W, Chang H, Lee S J, et al. Nat Mater, 2017, 16(5): 526 27992421 10.1038/nmat4825

[29] Zhang F F, Shang H, Wang L, et al. Adv Mater, 2021, 33(37): e2100866 34346090 10.1002/adma.202100866

[30] Mason J A, Sumida K, Herm Z R, et al. Energy Environ Sci, 2011, 4(8): 3030

[31] FitzGerald S A, Pierce C J, Rowsell J L, et al. J Am Chem Soc, 2013, 135(25): 9458 23711176 10.1021/ja402103u

[32] Geier S J, Mason J A, Bloch E D, et al. Chem Sci, 2013, 4(5): 2054

[33] Shivanna M, Otake K I, Zheng J J, et al. Chem Commun, 2020, 56(67): 9632 10.1039/d0cc03854g 32700696

[34] Tao Z R, Wu J X, Zhao Y J, et al. Nat Commun, 2019, 10(1): 2911 31266966 10.1038/s41467-019-10971-x PMC6606621

[35] Meng S S, Han T, Gu Y H, et al. Anal Chem, 2022, 94(41): 14251 36194134 10.1021/acs.analchem.2c02575

[36] Gao S B, Wang S C, Fan R H, et al. Biomed Pharmacother, 2020, 127: 110114 32304852 10.1016/j.biopha.2020.110114

[37] Barba C, Santa Maria G, Flores G, et al. J Agric Food Chem, 2010, 58(2): 752 20025225 10.1021/jf903156g

[38] Xie S M, Zhang Z J, Wang Z Y, et al. J Am Chem Soc, 2011, 133(31): 11892 21751804 10.1021/ja2044453

[39] Yang J R, Xie S M, Zhang J H, et al. J Chromatogr Sci, 2017, 54(9): 1467 27405508 10.1093/chromsci/bmw111

[40] Xie S M, Zhang X H, Zhang Z J, et al. Anal Bioanal Chem, 2013, 405(10): 3407 23361228 10.1007/s00216-013-6714-7

[41] Yang J R, Xie S M, Liu H, et al. Chromatographia, 2015, 78(7/8): 557

[42] Li L, Xie S M, Zhang J H, et al. Chem Res Chinese U, 2017, 33(1): 24

[43] Kou W T, Yang C X, Yan X P. J Mater Chem A, 2018, 6(37): 17861

[44] Wang B, Huang H L, Lü X L, et al. Inorg Chem, 2014, 53(17): 9254 25116469 10.1021/ic5013473

[45] Qin L Z, Xiong X H, Wang S H, et al. ACS Appl Mater, 2022, 14(40): 45444 10.1021/acsami.2c13446 36178410

[46] Gu Z Y, Jiang D Q, Wang H F, et al. J Phys Chem C, 2010, 114(1): 311

[47] Xu M, Cai P Y, Meng S S, et al. Angew Chem Int Ed, 2022, 61(37): e202207786 10.1002/anie.202207786 35723492

[48] Planas N, Mondloch J E, Tussupbayev S, et al. J Phys Chem Lett, 2014, 5(21): 3716 26278741 10.1021/jz501899j

[49] Carrasco S, Sanz Marco A, Martín Matute B. Organometallics, 2019, 38(18): 3429

[50] Gu X W, Wang J X, Wu E, et al. J Am Chem Soc, 2022, 144(6): 2614 35109657 10.1021/jacs.1c10973

[51] Liu D, Pei J Y, Zhang X, et al. Angew Chem Int Ed, 2023: 141795

[52] Yang S H, Lin X, Lewis W, et al. Nat Mater, 2012, 11(8): 710 22660661 10.1038/nmat3343

[53] Chang N, Gu Z Y, Yan X P. J Am Chem Soc, 2010, 132(39): 13645 20843033 10.1021/ja1058229

[54] Chen B L, Liang C D, Yang J, et al. Angew Chem Int Ed, 2006, 118(9): 1418

[55] Meng S S, Tao Z R, Tang W Q, et al. J Chromatogr A, 2020, 1632: 461604 33080532 10.1016/j.chroma.2020.461604

[56] Nugent P, Belmabkhout Y, Burd S D, et al. Nature, 2013, 495(7439): 80 23446349 10.1038/nature11893

[57] Suresh K, Aulakh D, Purewal J, et al. J Am Chem Soc, 2021, 143(28): 10727 34242007 10.1021/jacs.1c04926

[58] Marshall C R, Staudhammer S A, Brozek C K. Chem Sci, 2019, 10(41): 9396 32055316 10.1039/c9sc03802g PMC6979335

[59] Deria P, Mondloch J E, Tylianakis E, et al. J Am Chem Soc, 2013, 135(45): 16801 24175709 10.1021/ja408959g

[60] Wang T C, Vermeulen N A, Kim I S, et al. Nat Protoc, 2016, 11(1): 149 26678084 10.1038/nprot.2016.001

[61] Feng D, Gu Z Y, Li J R, et al. Angew Chem Int Ed, 2012, 51(41): 10307 10.1002/anie.201204475 22907870

[62] Xu M, Meng S S, Cai P Y, et al. Chem Sci, 2021, 12(11): 4104 10.1039/d0sc06747d PMC8179526 34163681

[63] Gu Z Y, Jiang J Q, Yan X P. Anal Chem, 2011, 83(13): 5093 21599025 10.1021/ac200646w

[64] Tang W Q, Zhao Y J, Xu M, et al. Angew Chem Int Ed, 2021, 60(13): 6920 10.1002/anie.202014673 33480119

[65] Abánades Lázaro I, Mazarakioti E C, Andres Garcia E, et al. J Mater Chem A, 2023, 11(10): 5320 10.1039/d2ta08934c PMC9990143 36911163

[66] Chen Y W, Wu H X, Yu L, et al. Chem Eng J, 2022, 431: 133284

[67] Wang Y, Huang N Y, Zhang X W, et al. Angew Chem Int Ed, 2019, 58(23): 7692 10.1002/anie.201902209 30913363

[68] Zhang F F, Shang H, Zhai B L, et al. AIChE J, 2023, 69(6): e18079

[69] Jiang Y J, Wang L Y, Yan T G, et al. Chem Sci, 2023, 14(2): 298 10.1039/d2sc05742e PMC9811657 36687342

[70] Xu M, Meng S S, Cai P Y, et al. ACS Cent Sci, 2022, 8(2): 184 35233451 10.1021/acscentsci.1c01344 PMC8874727

